# ECM Inspired Coating of Embroidered 3D Scaffolds Enhances Calvaria Bone Regeneration

**DOI:** 10.1155/2014/217078

**Published:** 2014-06-11

**Authors:** C. Rentsch, B. Rentsch, S. Heinemann, R. Bernhardt, B. Bischoff, Y. Förster, D. Scharnweber, S. Rammelt

**Affiliations:** ^1^Department of Trauma and Reconstructive Surgery, University Hospital Carl Gustav Carus, Technische Universität Dresden, Fetscherstraße 74, 01307 Dresden, Germany; ^2^Centre for Translational Bone, Joint and Soft Tissue Research, University Hospital and Medical Faculty, Technische Universität Dresden, Fetscherstraße 74, 01307 Dresden, Germany; ^3^Catgut GmbH, Gewerbepark 18, 08258 Markneukirchen, Germany; ^4^Max Bergmann Center of Biomaterials, Budapester Straße 27, 01069 Dresden, Germany; ^5^DFG-Center for Regenerative Therapies Dresden (CRTD), Fetscherstraße 105, 01307 Dresden, Germany

## Abstract

Resorbable polymeric implants and surface coatings are an emerging technology to treat bone defects and increase bone formation. This approach is of special interest in anatomical regions like the calvaria since adults lose the capacity to heal large calvarial defects. The present study assesses the potential of extracellular matrix inspired, embroidered polycaprolactone-co-lactide (PCL) scaffolds for the treatment of 13 mm full thickness calvarial bone defects in rabbits. Moreover the influence of a collagen/chondroitin sulfate (coll I/cs) coating of PCL scaffolds was evaluated. Defect areas filled with autologous bone and empty defects served as reference. The healing process was monitored over 6 months by combining a novel ultrasonographic method, radiographic imaging, biomechanical testing, and histology. The PCL coll I/cs treated group reached 68% new bone volume compared to the autologous group (100%) and the biomechanical stability of the defect area was similar to that of the gold standard. Histological investigations revealed a significantly more homogenous bone distribution over the whole defect area in the PCL coll I/cs group compared to the noncoated group. The bioactive, coll I/cs coated, highly porous, 3-dimensional PCL scaffold acted as a guide rail for new skull bone formation along and into the implant.

## 1. Introduction

The majority of cranial bone defects are caused by trauma, congenital deformity, or tumor resection. The skull bone has to be reconstructed to improve the functional and cosmetic outcome, correlating with the subsequent quality of life [1–3]. Since successful spontaneous healing only occurs in infants younger than two years, a variety of materials have been proposed to repair such defects, including autologous or allogeneic bone grafts, alloplastic materials, and tissue engineered bone scaffolds optionally seeded with cells or growth factors [2, 4]. Autologous bone grafts are the gold standard, but their clinical use is limited by donor site morbidity, availability, additional surgery, bone resorption at the recipient site, and difficulties with three-dimensional contouring [3, 4]. The most commonly used alloplastic materials are metals (e.g., stainless steel, titanium, gold, and aluminum), polymers (e.g., polymethyl methacrylate), and ceramics based on hydroxyapatite (HA). All metals, most ceramics, and many polymers are not considered to be biodegradable and therefore cannot be fully replaced by host bone tissue [5]. Foreign body reactions, stress shielding, and long term problems like infections, bone resorption, wound dehiscence, sunken bone flap, hematoma, and intraoperative hemodynamic instability are further issues [3]. The large amount of methods reflects that each technique has its own advantages and disadvantages as well as the need for new and improved treatment options [2].

Synthetic biodegradable scaffolds have been developed as an alternative for bone reconstruction. Implant materials based on calcium phosphate, biodegradable polymers, and composites, partly in combination with growth factors, bone marrow, or mesenchymal stem cells, are currently being studied as alternatives, but until now none of the synthetic bone graft materials has been generally accepted [6–11].

Natural or synthetic polymers can provide customized three-dimensional porous matrices that can temporarily support cells and guide their development [10–13].

The polyester of D,L-lactid, glycolid, or *ε*-caprolactone and their copolymers are approved by health authorities in various countries and commonly studied materials for biomedical applications in bone and cartilage repair [11, 14–19].

The polycaprolactone-co-lactide (PCL) used for this study was synthesized by ring-opening copolymerization of L-lactide and *ε*-caprolactone, with a molecular ratio of 75/25 (Gunze Ltd., Kyoto, Japan). Melt spinning of the material resulted in a resorbable, monofilament fiber, which is commercially available and approved as a medical device (PCL, surgical suture, Catgut GmbH, Markneukirchen, Germany) [14, 16, 20].

Embroidering, a traditional manufacturing technique, was used to produce PCL scaffolds allowing the control of their shape and size, the arrangement, and the orientation of the fibers. In addition, this technique is an effective tool to produce highly porous scaffolds that are required to allow cell ingrowth and an efficient transport of nutrients, oxygen, growth factors, and waste products through a rich vascularization. Despite these advantages, only few reports on this method are available [11, 18, 20–23].

Tissue engineering strategies include the transplantation of different kinds of cells alone or seeded on a variety of scaffolds and/or the use of biomolecules (growth factors, proteins, peptides, or polysaccharides), which affect the cells of the target tissue [24, 25].

In bone the organic extracellular matrix (ECM) consists of a highly ordered, site-specific network that is mainly composed of collagen type I (coll I) and smaller amounts of glycoproteins like fibronectin, proteoglycans like decorin and biglycan, and the glycosaminoglycans (GAGs) like chondroitin sulfate (cs), hyaluronan, dermatan, and heparan sulfate [26].

A promising approach to guide morphogenesis and tissue repair is mimicking the extracellular matrix to actively influence the cellular reaction and interaction with growth factors and cytokines [10, 27, 28]. A first step in the formation of an artificial ECM (aECM) is the immobilization of coll I to the surface of scaffolds or implants [20, 29–31].

The properties of bone implants can be further improved by the addition of GAGs like cs [11, 28, 32–35]. CS plays a key role in bone development, remodeling, and healing by interacting with other molecules of the ECM, mediating cell adhesion, and providing the binding of different growth factors or cytokines on the ECM [11, 20, 28, 33, 36, 37]. Several* in vitro* and* in vivo* studies on long bones have demonstrated that embroidered PCL scaffolds biologically modified with coll I/cs provide an appropriate network of interconnecting pores to act as a temporary matrix for cell adhesion, migration, proliferation, and differentiation [11, 18, 20, 21, 23].

In the light of these results, the present study was designed to assess the healing capacity of the bioactive, coll I/cs coated, highly porous, 3-dimensional PCL scaffolds as skull bone implants.

During skeletal formation the calvarial bone involves a process known as intramembranous ossification (cartilage is not present) which is different from endochondral ossification processes in long bones. Compared to long bones, calvarial bone is more biological inert due to its reduced blood supply. It has to be considered that calvarial bone lacks muscle enclosures so the blood supply is less than in long bones. According to that it is even more important to reach a good scaffold vascularization in animal experimental investigations.

The aim of this study was to verify the design of the implant as skull bone implant. Additionally, the bone healing quality in a mechanically unloaded bone defect, the performance of the implant material, the status of inflammation, and vascularization were evaluated.

To achieve this goal four groups (*n* = 8) of randomly divided New Zealand white rabbits were treated with either noncoated or coll I/cs coated PCL scaffolds. Untreated defects and defects treated with autologous bone grafting, as the current clinical gold standard, served as controls. New bone formation was determined using ultrasound as life imaging method as well as by radiological, computer tomographical, biomechanical, and histological investigations.

## 2. Materials and Methods

### 2.1. Production and Coating of the PCL Scaffolds

The polycaprolactone-co-lactide was synthesized of L-lactide and *ε*-caprolactone, with a molecular ratio of 75/25 (Gunze Ltd., Kyoto, Japan), melt spun, and resulted in a resorbable, monofilament fiber (PCL, surgical suture, Catgut GmbH, Markneukirchen, Germany). The textile scaffolds were made on a computer aided embroidery machine and had a triaxial structure with a stitch length of 1.4 mm and a mesh spacing of 1.2 mm. The polyvinyl alcohol (ground fabrics) was removed from the embroidered scaffolds by washing them with water and the protecting glaze was washed from the scaffolds with n-heptane. Afterwards the scaffolds were treated with 1 M NaOH in 50% methanol for hydrophilization of the scaffold surface, washed with water, dried, and finally sterilized with ethylene oxide [20].

The PCL scaffolds were coated with the coll I/cs matrix using a dip coating process. Porcine skin coll I (MBP GmbH, Neustadt-Glewe, Germany) was suspended in 0.01 M acetic acid, diluted to 2.5 mg/mL in phosphate buffer (10 mM KH_2_PO_4_, 50 mM Na_2_HPO_4_, pH 7.4) with 1.25 mg/mL porcine cs A (cs of porcine trachea, Kraeber & Co GmbH, Ellerbek, Germany). Coll I fibrils were adsorbed on the scaffold surface, whereas cs was immobilized within the collagen matrix after an incubation of 2 h at 37°C. The scaffolds were washed twice with water, dried, and finally sterilized with ethylene oxide. The characterization of the scaffold coating and the cell response was previously described by Rentsch et al., [20].

### 2.2. Scaffold Design

Scaffolds for the skull defect had a size of 13 mm in diameter and a total thickness of 3 mm according to that of the calvarial bone. An overlapping lid of 19 mm in diameter and 1 mm thickness was added to prevent subsidence. Both embroidered scaffold parts (main scaffold and lid scaffold) were made and sewn together on electronically guided machines (Möckel embroidery and engineering company, Auerbach, Germany). The 13 × 3 mm part of the scaffold was placed within the calvarial defect area. The 19 mm in diameter lid fixed the implant in place due to the contact of the surrounding skull bone.

### 2.3. Microcomputer Tomography (*μ*CT) of the Scaffolds

For a nondestructive *μ*CT analysis a Scanco vivaCT 75 system (Scanco Medical, Brüttisellen, Switzerland) was used. The samples were measured by radiological energy of 55 keV and 1500 projections. The voxel resolution of the reconstructed volume was 20 *μ*m. The porosity and the pore size distribution were measured with the Scanco evaluation software.

### 2.4. Scanning Electron Microscopy (SEM)

Samples were mounted on stubs, coated with a 50 nm gold layer (Leica EM SCD 005, Leica Microsystems GmbH, Vienna, Austria), and scaffolds were observed in a XL30 FEG ESEM (Philips, Eindhoven, Netherland) in a HiVak mode with acceleration voltages of 2–10 kV.

### 2.5. Animal Experiments

The study has been licensed by the regional veterinary board (24-9168.11-1/2009-5). All animals were cared for according to the European guidelines for the care and use of laboratory animals (Directive 24.11.1986, 86/609/CEE). A total number of 32 New Zealand white rabbits (female, on average 3 kg, Charles River Laboratories, Sulzfeld, Germany) were divided randomly into the following 4 groups of 8 animals: empty control group; autologous bone group (representing the clinical gold standard); PCL noncoated group; and PCL coll I/cs coated group.

**Figure 1:**
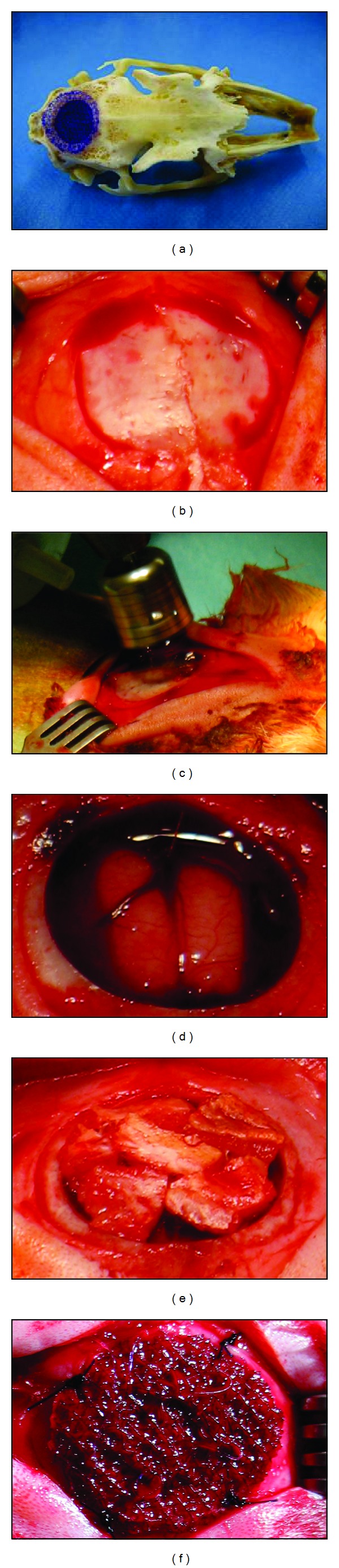
Surgical procedure. (a) Representing photograph showing the scaffold and the implantation site (defect) in a macerated rabbit skull. (b) Intraoperative situs of the cranium after removal of the periost in a diameter of 15 mm. (c) Creation of the defect by trephine drilling. (d) Untreated defect. (e) Autologous bone pieces were fixed with fibrin glue. (f) Defect filling with a PCL scaffold.

The rabbits were anaesthetized with a combination of ketamine (35 mg/kg body weight, Kemint, Alvetra GmbH, Neumünster, Germany) and xylazine (5 mg/kg body weight, Rompun, Bayer, Germany). The surgical sites were depilated using depilatory cream (Veet GmbH, Mannheim, Germany) and disinfected (Cutasept G, Bode Chemie GmbH, Hamburg, Germany). A sagittal incision was made at the skull along the midline from the frontal to the occipital bone. The periosteum was resected in a diameter of 15 mm and a 13 mm full thickness defect bone was created carefully in the central parietal skull with a dental trephine (L10 mm, D13 mm, Meisinger, Neuss, Germany) with continuous irrigation of sterile saline (NaCl 0.9%, Fresenius GmbH, Bad Homburg, Germany). The cranial cap was removed and care was taken to prevent damage to the dura (Figures 1(b)–1(d)).

The defect in the autologous bone group was filled with preserved fragmented autologous skull bone. Therefore, the removed cranial cap was split into 8–10 bone fragments using surgical tongs. The bone pieces were placed upside down directly onto the dura covering the defect area as good as possible, followed by a fixation with fibrin glue (Tissucol Dus S, Baxter GmbH, Unterschleißheim, Germany) to prevent migration of the bone pieces. PCL groups received either noncoated or coll I/cs coated implants (Figures 1(e) and 1(f)). Finally, the soft tissue was folded back and closed with a nonresorbable suture (Mariderm, Catgut GmbH, Markneukirchen). A single-shot antibiotic prophylaxis (15 mg/kg body weight, Duphamox, Pfizer GmbH, Berlin, Germany) was administered and Carprofen (1.4 mg/kg body weight, Rimadyl, Pfizer GmbH, Berlin, Germany) was given immediately and 24 hours after surgery for pain prevention. The healing process was monitored using ultrasonography. After 6 months all animals were anesthetized (ketamine/xylazine mixture) and euthanized with a combination compound of 200 mg Embutramide, 50 mg Mebezonium, and 5 mg Tetracaine per 1 mL (0.3 mL/kg body weight i.v., T61, Intervent GmbH Unterschleißheim, Germany). The entire cranial vault was carefully removed from each animal with an oscillating saw and all samples were fixed in 4% formalin (SAV LP GmbH, Flintsbach, Germany) until further analysis.

### 2.6. Tracking of New Bone Formation via Ultrasound

**Figure 2:**
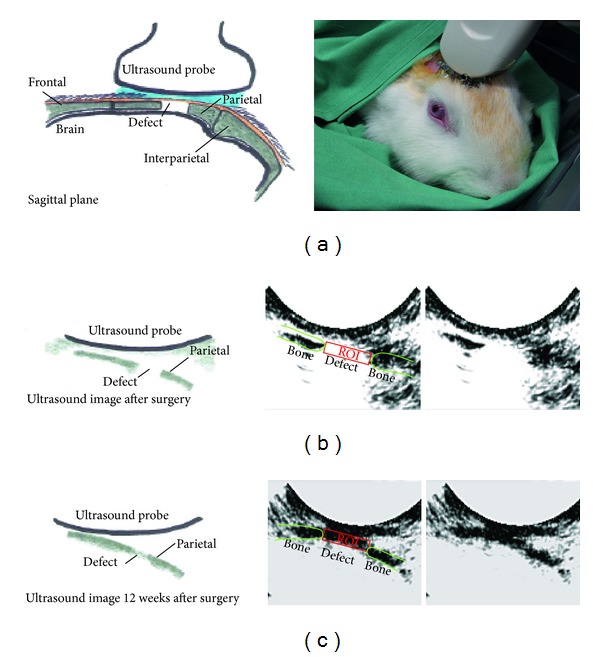
Schematic presentation of ultrasonographic examination of the rabbit skull and image analysis. (a) Defect location and ultrasound probe positioning during examination. (b) Schematic drawing (left) and corresponding ultrasound image (right) of an empty defect 24 h after surgery. (c) Schematic drawing (left) and ultrasound image (right) of a PCL coll I/cs coated scaffold at 12 weeks after surgery with evident new tissue formation. A defined ROI (red square) was used to quantify the tissue formation within the defect area and both parietal bone ends are marked in green.

A PC-sonographic system (taberna pro medicum GmbH, Lüneburg, Germany) containing a TELEMED eco blaster 128 (TELEMED, Vilnius, Lithuania) and a C3.5/20/128 sensor was used for imaging the defect at 24 hours and 6 and 12 weeks following surgery (Figure 2). The following measurement parameters were defined: a frequency of 3 MHz, 53 frames per second, depth of 90 mm, averaging 8 images, and a dynamic of 72 dB. Rabbits were placed in a small animal care box and covered with a blanket; thereby no anesthesia was necessary during the examination. Approximately 5 mL of ultrasound gel (Dahlhausen & Co. GmbH, Köln, Germany) was applied onto the skull. Three images were obtained for each animal per group and time point. Images were quantified by ImageJ (http://rsb.info.nih.gov/ij/) using a defined region of interest (ROI) (Figures 2(a), 2(b), and 2(c)). The gray scale picture was transferred to an 8 bit image presenting a color range between 0 (white) and 255 (black). After applying a threshold frame of 188–255, all black pixels per area of ROI were quantified.

### 2.7. Radiographic Imaging

Plain radiographs were taken of all explants with a mobile X-ray unit (AMX 4, GE Healthcare, Buckinghamshire, UK) using 52 kV and 2.5 mAs.

### 2.8. Microcomputed Tomography (*μ*CT) Analysis of the Explants

All explants of each group were assessed with a nondestructive *μ*CT analysis using the Scanco vivaCT 75 systems (Scanco Medical, Brüttisellen, Switzerland). The explanted and fixed samples were measured with X-ray energy of 70 keV and 500 projections. The voxel resolution of the reconstructed images was 70 *μ*m. The quantification of the new bone volume within the defect area was done using 55 sagittal *μ*CT images (8 animals/group) with an image distance of 0.236 mm each. A region of interest (ROI) was defined and the pixel/area was quantified using ImageJ (Figure 6(c)). The bone volume within the ROI of each group was then expressed as a percentage of the total defect volume of nonoperated calvarial bone (not shown). The latter was set to 100%.

### 2.9. Biomechanical Testing

Push-out tests of the tissue-engineered calvaria and control samples were performed to evaluate the mechanical integrity of the defect area. An Instron 5566 uniaxial testing system (Instron, Pfungstadt, Germany) equipped with a 10 kN load cell and a flat 10 mm diameter indenter fitting centered in the defect area was used. An initial load of 1 N was applied, followed by constant displacement at a strain rate of 0.9 mm/min until implant failure. Force and displacement were recorded simultaneously. Relative stiffness of the implant site was calculated as a quotient of ultimate force and corresponding displacement. Four samples were tested for each group.

### 2.10. Histology 

A 2 × 2 cm sample centered around the defect was cut out of the rabbit calvarium. The samples were washed, decalcified for 1 week in ethylenediaminetetraacetic acid (OSTEOSOFT, Merck KGaA, Darmstadt, Germany; to adjust pH to 7.4–7.6 use NaOH) using an automated microwave based tissue Processor RHS-1 (Diapath S.p.A, Martinengo, Italy), and dehydrated overnight in a Thermo Scientific STP 420ES Tissue Processor (Microm International GmbH, Walldorf, Germany). Each sample was divided into two equal parts and one was embedded in a horizontal and one in a vertical orientation in methyl methacrylate (Technovit 9100 N, Heraeus Kulzer GmbH, Werheim, Germany). Sections of 3 *μ*m of were prepared with a rotation microtome RM2055 (Leica Microsystems, Wetzlar, Germany) and the methyl methacrylate was removed using twice xylene for 20 min, twice 2-methoxy ethyl acetate for 20 min, twice acetone for 5 min, and 80% ethanol. Samples were stained for light microscopy (Leica DMRBE Research Microscope, Camera Leica DC300, Leica Microsystems, Wetzlar, Germany) with Haematoxylin & Eosin (HE), (VWR, International GmbH, Darmstadt, Germany) and Masson-Goldner trichrome staining (Masson-Goldner trichrome staining kit, Merck KGaA, Darmstadt, Germany). Finally, sections were mounted in Canada balsam solution (Sigma-Aldrich Chemie GmbH, Munich, Germany). Four samples were tested for each group. Three histological sections of each animal (both section planes) were evaluated individually.

### 2.11. Data Analysis

All statistical analyses were done using the Student's *t*-test.

## 3. Results

### 3.1. *μ*CT and SEM of PCL Scaffolds

**Figure 3:**
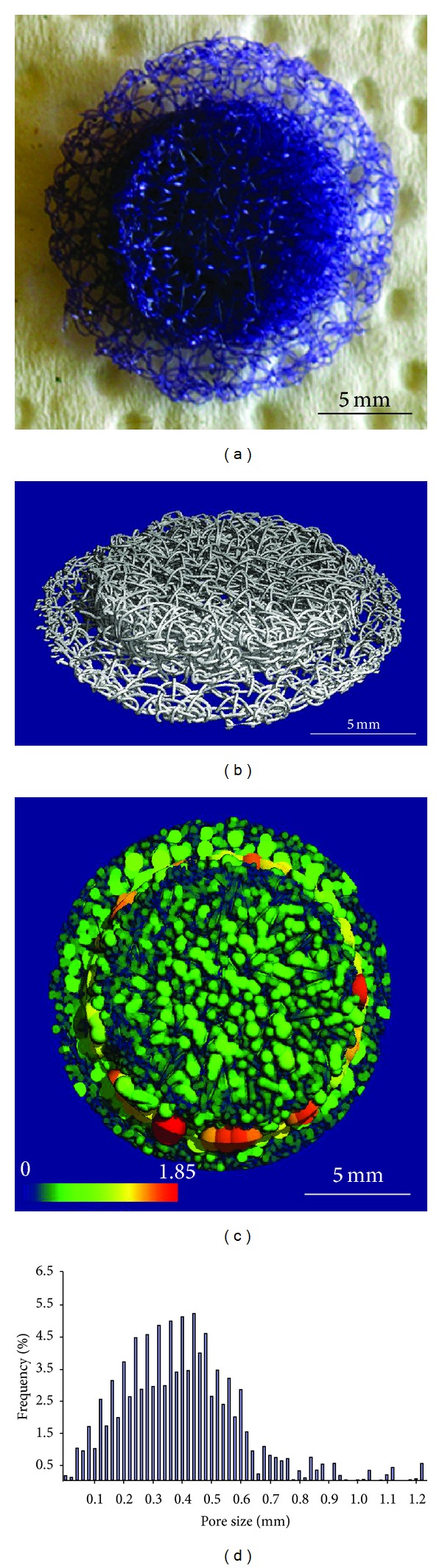
Characteristics of the embroidered scaffold designed for calvarium reconstruction. (a) Photograph and (b) three-dimensional *μ*CT reconstruction of the embroidered scaffold showing an open porosity of 87%. ((c) and (d)) The analysis of the pore size distribution (Scanco vivaCT 75 system) showed homogeneously interconnected pores ranging between 0.06 and 0.7 mm distributed over the whole stack.

Scaffolds for the skull defect had a size of 13 mm in diameter and a total thickness of 3 mm according to that of the calvarial bone, including an overlapping lid of 19 mm to prevent subsidence (Figures 3(a) and 3(b)). Scaffold analysis, using *μ*CT, revealed an open porous network of 87% with a pore size of 0.06–0.7 mm (Figures 3(c) and 3(d); Table 1).

**Figure 4:**
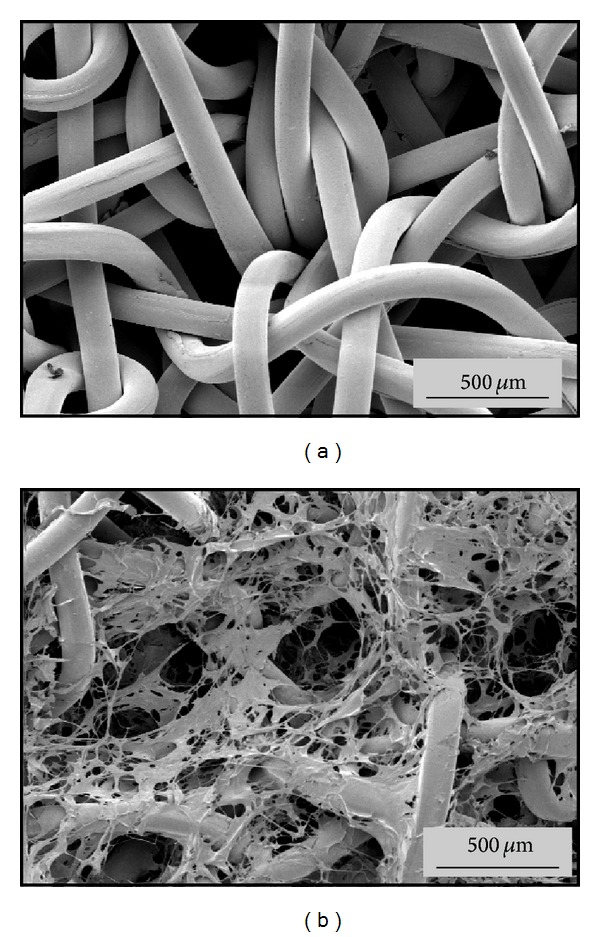
SEM micrographs of the embroidered PCL scaffolds. (a) Noncoated PCL scaffold. The triaxial structure had a stitch length of 1.4 mm and a mesh spacing of 1.2 mm. (b) PCL coll I/cs coated scaffold. The coating covers the polymer fibers and partly fills the pores.

Figure 4 presents an SEM image of the PCL fibers within the 3-dimensional scaffold (Figure 4(a)). After surface coating with coll I/cs an additional network of extracellular components was presented to the new ingrowing tissue (Figure 4(b)).

### 3.2. Surgical Procedure

All 32 animals survived the surgery and the following 6 months without any complications.

### 3.3. Tracking of Tissue Formation within the Defect Area with Ultrasonography

**Figure 5:**
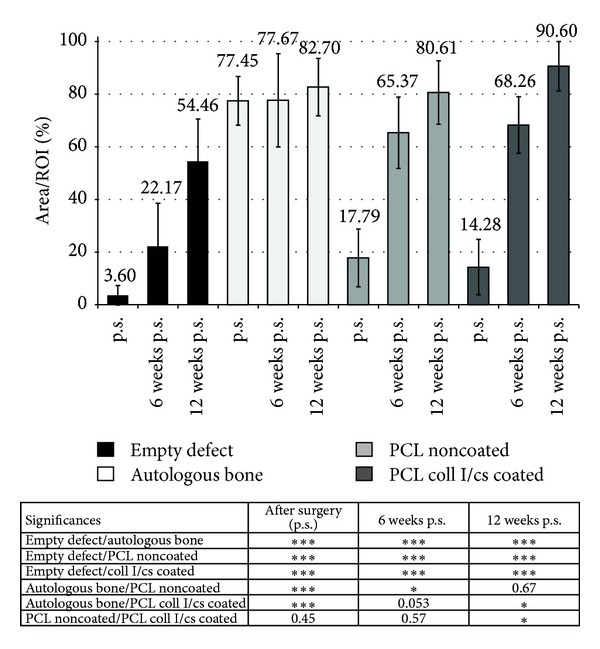
Tracking of new tissue formation with ultrasonography. Significances as indicated: *P < 0.05, **P < 0.01, ***P < 0.001 (Student's *t*-test).

The 13 mm skull defect could be detected reliably via ultrasound after surgery and new bone formation could be followed up to 12 weeks (Figure 5). The empty defect showed the lowest tissue formation compared to all other groups over all time points of measurement. Within the 12 weeks a significant increase of tissue formation could be measured (3.6%, 22.2%, and 54.5%). The group containing autologous bone showed a permanent tissue volume of around 80% over all time points. According to that fact, possible healing processes within bone fragments could not be detected via sonification. The PCL noncoated and PCL coll I/cs coated group showed a higher ultrasound reflecting matrix (17.8% and 14.3% after surgery) in the defect area compared to the empty control group (3.60% after surgery). After 6 weeks, the PCL noncoated and PCL coll I/cs coated groups presented four times as much (70%) matrix within the defect area compared to the postsurgery measurements, indicating a matrix forming process. Finally, the PCL coll I/cs coated group showed significantly more (90.6%) detectable tissue than the PCL noncoated group (80.6%).

### 3.4. Radiographic and *μ*CT Analysis of the Explants

**Figure 6:**
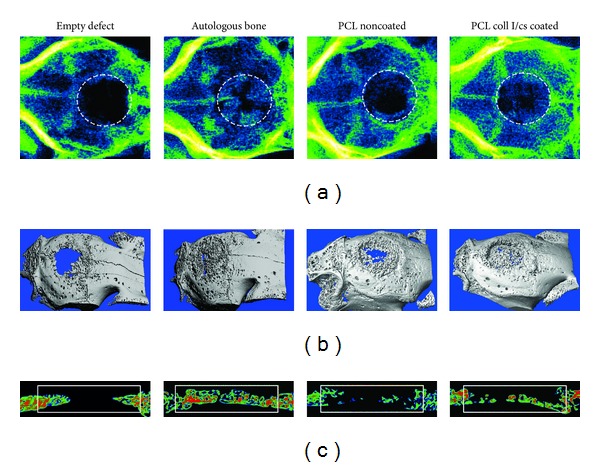
Radiography, 3-dimensional *μ*CT reconstruction, and *μ*CT bone quantification. (a) Radiographs taken after skull preparation (false coloring). The defect area is marked with a white circle. (b) Image of a 3-dimensional *μ*CT reconstruction. (c) Sagittal section plane of the defect area. The white frame presents the ROI for bone quantification. One representative image of one animal from each group is shown.

Six months after surgery new bone formation could be visualized in all animals of each group in plain radiographs (Figure 6(a)) and *μ*CT (Figure 6(b)). The empty defect group showed new bone formation predominantly at the margin of the defect. All reimplanted bone fragments of the autologous bone group were reconnected to the skull bone or among themselves, but no new homologous bone formation occurred. Single fragments could still be detected. Both implant groups showed a higher new bone formation compared to the empty group. The PCL coll I/cs coated scaffolds showed a more homologous new bone formation across the whole defect area compared to the PCL noncoated implants. Cross sections for bone quantification confirmed these findings (Figure 6(c)).

### 3.5. Quantification of New Bone Formation (*μ*CT Analysis)

**Figure 7:**
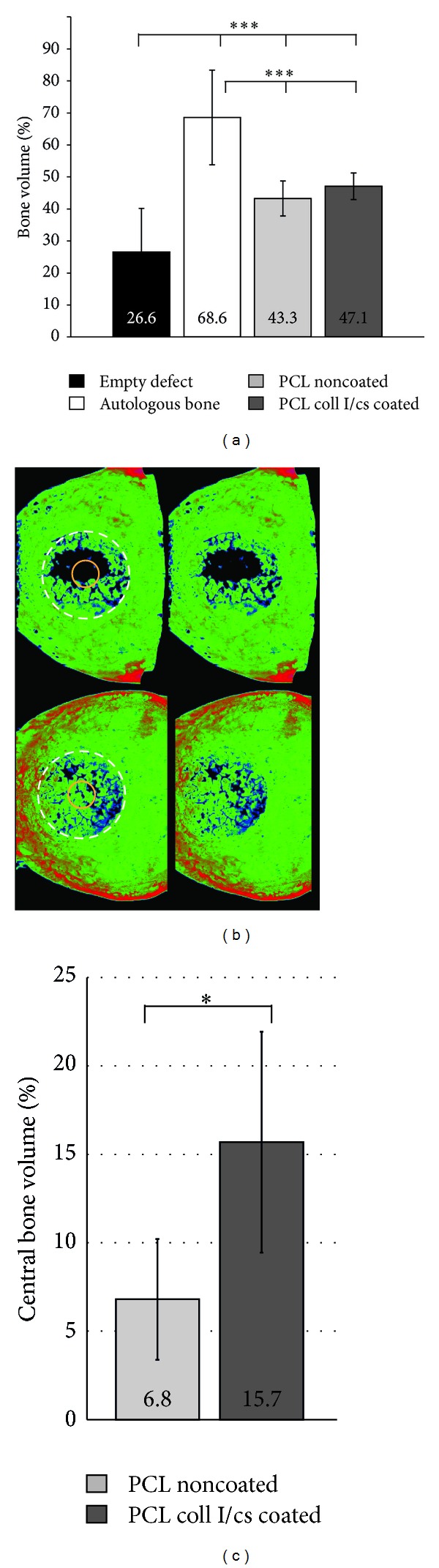
Quantification of bone volume within the defect area. (a) Quantification of bone volume related to the entire bone defect area of 13 mm. (b) Representative images of PCL noncoated (top) and PCL coll I/cs coated (bottom) 3-dimensional *μ*CT reconstructions. The left images are marked for visualization and right picture presents the original. The white circles in the left images represent the whole defect area and the yellow circles the 4 mm central part. (c) Quantification of bone formation in the central defect area. Significant differences as indicated: *P < 0.05, ***P < 0.001 (Student's *t*-test).

Based on the *μ*CT quantification of newly produced bone in the defect area, the PCL coll I/cs coated group (47.1%) showed the largest amount of new bone formation compared to PCL noncoated (43.3%) and the empty defect (26.6%) group. Bone volume averaged 68.6% in the autologous group (Figure 7(a)).

Detailed analysis of the whole defect area revealed a more homogenous bone distribution within the PCL coll I/cs coated scaffold group compared to the PCL noncoated group (Figure 7(b)). The central area of PCL coll I/cs coated scaffolds showed more than twice as much bone (15.7%) as the PCL noncoated scaffolds (6.8%) (Figure 7(c)). This difference was statistically significant.

### 3.6. Biomechanical Testing

**Figure 8:**
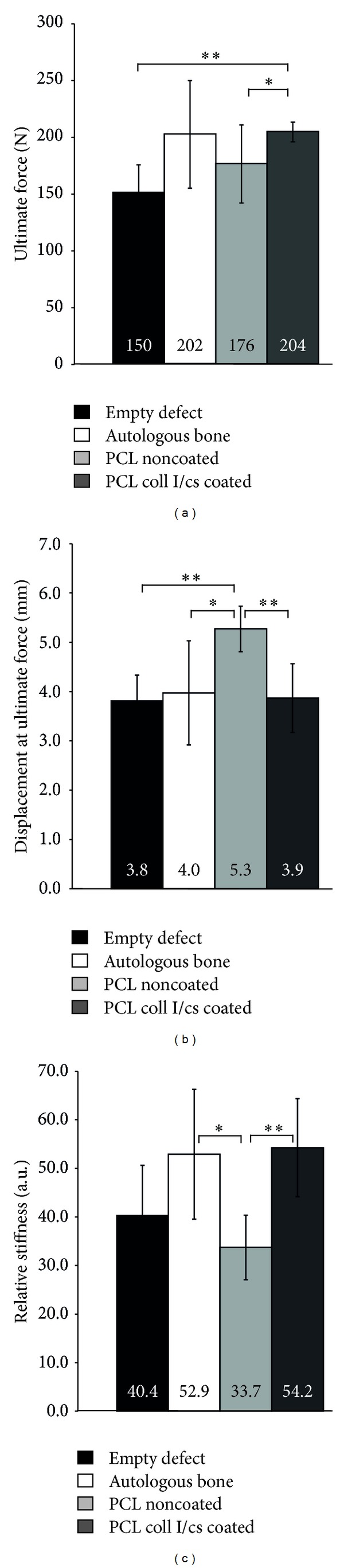
Biomechanical evaluation. (a) Ultimate force in newton (N). (b) Displacement of ultimate force in millimeter (mm). (c) Relative stiffness. Significant differences as indicated: *P < 0.05, **P < 0.01, ***P < 0.001 (Student's *t*-test).

The push-out test was performed in order to evaluate the mechanical integrity of the scaffold reconstructed calvaria region. The empty defects and autologous bone treated defects served as a reference. In all tests the push-out of the reconstructed area was observed to be the dominant type of failure whereas the host calvaria maintained its integrity. Statistical analysis of characteristic values obtained from the force/displacement curves is shown in Figures 8(a) and 8(b). The ultimate force recorded during complete push-out was the lowest for the empty defects (150 N) and the highest for the PCL coll I/cs coated treated defects (204 N) whereby they reached autologous bone level (202 N).

Regarding the relative stiffness of the reconstructed area, the results also demonstrate the significant influence of the biomaterial and its coating to achieve a bone like stability. Whereas PCL noncoated showed no effect, the relative stiffness was nearly 60% higher for PCL coll I/cs coated and therefore similar to the gold standard (Figure 8(c)).

### 3.7. Histology

**Figure 9:**
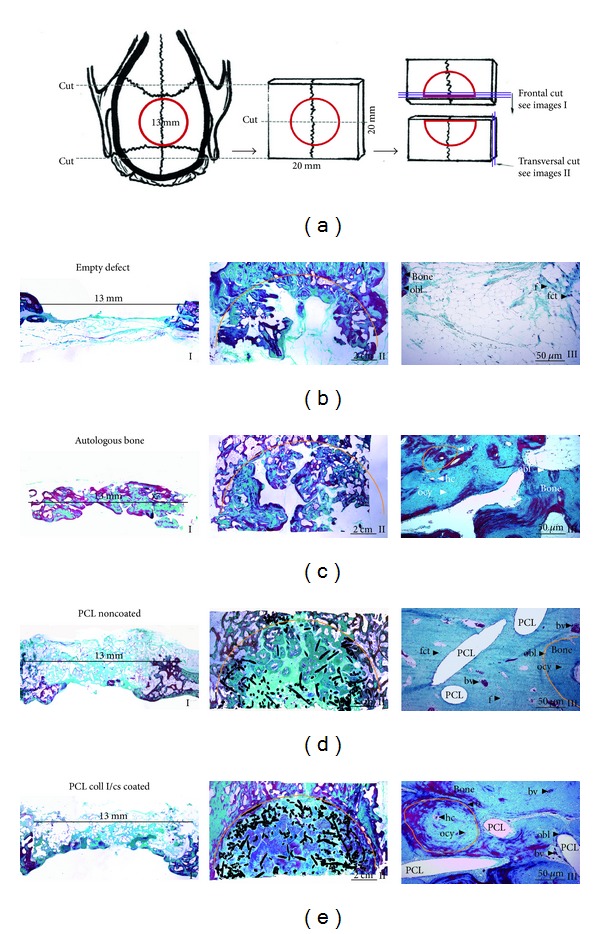
Representative histological images of one animal from each group (Masson-Goldner trichrome staining). (a) Schematic drawing of the histological sample preparation. (b) Empty defect. (c) Autologous bone. (d) PCL noncoated. (e) PCL coll I/cs coated. I: frontal plane with the defect area marked by the 13 mm line (1x). II: transversal plane with the defect area is marked by the orange semicircle (1x). PCL scaffold is marked in black. III: enlarged section of the defect zone (20x). obl: osteoblast, ocy: osteocyte, o: osteon (orange), hc: Haversian canal, f: fibroblast, bv: blood vessel with erythrocyte, and fct: firm connective tissue.

Masson-Goldner trichrome staining presented bone in turquoise/red color, whereas fibrous tissue is presented in light turquoise color. All groups showed new bone formation within the 13 mm defect (Figure 9). The empty group presented new bone formation at the margins without bridging the defect. The central part of the defects was filled with fibrous tissue (Figure 9(b)). All implanted autologous bone fragments of the autologous group were vital and reconnected to the skull bone or among themselves, but no homogeneous new bone formation occurred. Single bone fragments could still be detected and empty spots were still measurable after 6 months of implantation (Figure 9(c)).

Both PCL groups showed newly formed lamellar bone inside the scaffolds presenting osteons, including Haversian canals (see orange circle in Figures 9(d) and 9(e) III). Bone formation occurred mostly at the lamina interna (located next to the dura mater) Figures 9(d) I and 9(e) I). The scaffolds were completely vascularized (see blood vessel (bv) in Figures 9(d) and 9(e) III) and no area appeared completely free of tissue. The PCL scaffolds still existed 6 months after implantation. No signs of a chronic inflammatory reaction like accumulation of granulation tissue, lymphocytes, macrophages, or foreign body giant cells were visible around the implant material. The PCL coll I/cs coated group showed a higher amount of new bone formation within the central defect as compared to the PCL noncoated group (Figure 9(e) II).

## 4. Discussion

The aim of this* in vivo* study was to characterize the polycaprolactone-co-lactid (PCL) scaffold as a skull bone implant and to evaluate the effect of surface coating with colI I/cs on these scaffolds in relation to untreated defects and defects filled with autologous bone. Parameters for evaluation were based on ultrasonographic and radiological investigations, biomechanical testing, and histology.

The PCL material used in this study describes a copolymer made of polycaprolactone-co-lactid (see Section 2). The commercially available copolymer suture is sold under this (PCL) trade name from the Catgut GmbH and therefore the abbreviation PCL is used in this publication. To avoid confusion with polycaprolactone, also described as PCL, which is not copolymer, the following discussion will refer to polycaprolactone.

### 4.1. Animal Model and Defect Size

Various types and sizes of calvarial bone defects are described in rabbits [38–45]. In the present study, the defects could be created safely between the frontal and interparietal skull bone without touching the coronal or lambdoid suture in order to avoid as much fibrous suture tissue within the defect as possible. To create a defect with the same thickness of bone on either side of the sagittal suture at the parietal bone anatomical conditions were not given. Resulting, a circular skull defect of 13 mm diameter was chosen and placed centrally within the parietal bone.

To fulfil the definition of a critical size defect (CSD), less than 10% of bone formation within the CSD should be observed during the lifetime of the animal [46]. Since the empty defect showed 26.6% new bone formation after 6 months, but no bridging, the defect size does not fulfil this definition of a CSD.

According to data available from the literature (Table 2), new bone formation within a 15 mm defect ranges from 17.5 to 24.4% after 12 weeks [38, 42, 44]. In contrast, Schantz et al. discovered only 1.2% new bone formation after 12 months and Kroese-Deutman et al. mentioned that all 15 mm defects in their study were open after 12 weeks but did not provide bone volume data for this group [39, 45]. While the available data do not allow a precise definition of a CSD in the rabbit calvaria, the amount of newly formed bone within the 13 mm defect at 6 months in our study is comparable to most of the previous studies [38, 42, 44]. The sparse amount of new bone formation from the margins makes the untreated defects reliable as a control group.

### 4.2. Bone Quantification within the Defects

In the present study, the PCL scaffolds were successfully integrated into large bone defects. PCL noncoated and PCL coll I/cs coated scaffolds showed significantly more (43.3 and 47.1%) new bone formation within the defect area compared to the empty defects (26.6%) after 6 months. The distribution of new bone formation within the defect area was more uniform with significantly more new bone in the central portion of the coll I/cs coated scaffold compared to the noncoated PCL scaffold (Figure 7(c)).

An extensive review of the literature revealed 26 studies on the repair of rabbit skull defects. Eight studies presented the final amount of new bone formation within the defect in percentage and therefore seemed to be appropriate for comparison (Table 2). However, these comparisons are limited because of strong variations in defect size, time points, implant materials, and the number of investigated animals.

It becomes obvious that skull implants made of different polymers like polycaprolactone, poly(lactic-co-glycolic acid) (PLGA), tyrosine-derived polycarbonate (TyrPC), or tricalcium phosphate (TCP) derivative resulted in considerably less new bone formation within the defects than the PCL used in the present study (Table 2). The amount of new bone formation ranged from 1.2% for medical grade polycaprolactone [45] to 16.0% for TyrPC [43], 25.1% for PGLA [40], and 32.0–34.9% for composites made of PLGA/TCP or polycaprolactone-TCP [40, 41]. Biologization of medical grade polycaprolactone scaffolds with bone marrow or osteoblasts increased the bone volume up to 12.5% or 14.1% [45] but never reached the amount of new bone formation in the present study (43.3% for PCL noncoated and 47.1% for PCL coll I/cs coated).

The application of bone morphogenetic protein (BMP-2) resulted in a considerable increase of the amount of new bone formation within TyrPC (34.0%), biocomposite (BC: 27.0%), and calcium phosphate cement (CPC: 45.8%) scaffolds [38, 43, 44]. These BMP-2 induced bone quantities come into the range of the new bone formation using PCL noncoated or PCL coll I/cs coated scaffolds without any growth factor application, indicating that the PCL scaffold by itself acts beyond osteoconductive properties. Although growth factor applications are used for animal tissue engineering studies, the clinical use of BMP is limited by the cost, rapid degradation* in situ,* and inconsistent biological activity [47, 48].

The superior biological performance of the PCL scaffolds used in our study could be explained by the copolymer composition which comes along with a compatible degradation rate and degradation products [14, 17]. The used PCL fiber shows a degradation rate of around 25 weeks via hydrolysis at* in vitro* as well as* in vivo* investigations [14]. The PCL material used in this study was still visible after 6 months of implantation. This is not unexpected, given that the extent and the mechanism of the polymer hydrolysis are depending on the amount and the location of water molecules [49].

The PCL scaffold provides an appropriate network of material and interconnecting pores to act as a temporary matrix for new bone formation similar to autologous bone. The structure allows cell penetration and proper vascularization which is needed for bone formation. Compared to long bones, calvarial bone is more biological inert due to its reduced blood supply. It has to be considered that large areas of human skull bone are lacking muscle insertions so the blood supply to the human calvaria is even less than in other mammals [50]. According to that it is even more important to reach a good scaffold vascularization in animal experimental investigations. The importance of blood vessel formation for intramembranous ossification in cranial bones and bone repair is also described by Kanczler and Oreffo [51]. The vasculature transports oxygen, nutrients, and different cell types within the implant and supports tissue formation [51]. In this study the bone formation within the PCL scaffold occurred mostly at the lamina interna indicating a better nutrition and blood supply from the side of the dura mater compared to the scalp side.

The surface coating with components of the ECM (coll I/cs) further increased the amount of new bone formation within the scaffolds. This leads to the conclusion that the coll I/cs coating tents to an increased surface area for cell binding and growth factor adsorption and as a result of a more homogenous bone distribution within the PCL coll I/cs coated scaffold. This is in accordance with earlier* in vivo* and* in vitro* studies that have shown similar effects in several cell culture experiments and in long bone defects in small and large animal models [11, 18, 20, 21, 23].

The effect of collagen coatings may be based on the interaction with osteoblastic cells via integrin receptors. Glycosaminoglycans (GAGs) like chondroitin sulfate or dermatan sulfate are important components of the bone ECM and play a key role in bone development, remodeling, and regeneration by interacting with other ECM molecules, growth factors, cytokines, and cells [11, 18, 20, 21, 23, 28, 32, 33, 36, 37, 52, 53]. GAGs are known to specifically bind growth factors and modulate their activity. This is due primarily to an interaction of the GAGs negatively charged sulfate groups with positively charged amino acid sequences of the growth factors and has been reported among others, for fibroblast growth factor (FGF) and transforming growth factor beta, (TGF-*β*) [54].

### 4.3. Tracking of Tissue Formation within the Defect Area with Ultrasonography

*In vivo* tracking of new tissue formation within large defects can be done by life computed tomography (CT) imaging [55] because standard radiographic methods are not applicable due to the three-dimensional structure of the rabbit skull. However, life CT is expensive and often not available in animal care units, especially for animals larger than rats. Additionally, the animals have to be anesthetized for each investigation and so the risk of death is increasing during anesthesia stress.

The skull defect was easily accessible with an ultrasound probe and the defect was clearly detectable after surgery and over the further course. The obtained data roughly match those from the postmortal *μ*CT. The authors conclude that ultrasonography can be used as a method to track tissue formation within large calvarial bone defects. Because ultrasound is not associated with radiation it is of potential clinical use. However, the method has several limitations. The given setup could not distinguish between new bone and new firm connective tissue formation because the ultrasonic waves were reflected in a similar manner by both tissues. Furthermore, for skull defects, this method can only be used for implants with a very low ultrasound reflecting matrix like PCL.

Wefer et al. established an ultrasound method on sheep long bones to predict the healing of a defect filled with a bone graft substitute or cancellous bone graft. They also come to an agreement that a follow-up study for bone healing using sonography is possible, but the bony integration inside of the implant cannot be given definitely [56].

### 4.4. Clinical Outlook

The PCL scaffold seems to be a promising bone implant for mechanically unloaded defect filling like skull defects. It can be provided in any size and shape due to the embroidery technique. The scaffold design allows the fixation of the implant by suturing the scaffold lid to the surrounding periost tissue so no implant movement occurs. Even the scaffold does not provide initial mechanical stability like autologous bone; the 3-dimensional structure protects the underlying brain tissue in first place. The PCL material degrades in a biologically relevant timeframe of 6 to 12 months; meanwhile new bone formation can occur. The applied PCL scaffold could be additionally fixed by metal nets to provide a stronger mechanical stabilization in case of larger defects in human.

## 5. Conclusion

This work has demonstrated that embroidered PCL scaffolds can act as skull bone implants for large defects in rabbits. The implant design and the material allowed good surgical handling and a high amount of new bone formation within the scaffold after six months. The combination of scaffold and bioactive surface coating (coll I/cs) enhanced new bone formation and led to a more homogeneous distribution of newly formed bone within the scaffolds. The use of these scaffolds finally resulted in a homogeneous bridging of the defect with high-quality bone that was histologically and biomechanically similar to autologous bone.

## Figures and Tables

**Table 1:** Characteristics of the embroidered scaffolds designed for calvarial defects in rabbits.

Scaffold disk thickness (mm)	Diameter (mm)	Weight (mg)	Porosity (%)	Mean pore size (*μ*m)	Range pore size > 1% (*μ*m)
Total 4		107.2 ± 7.2	87	425	60–700
Overlapping lid 1	Overlapping lid 19				
Basis scaffold 3	Basis scaffold 13				

**Table 2:** Literature analysis.

Publication	Animal/number/group	Defect size/time of study	Implant	Bone volume in %
[38]	Rabbit/*n* = 10	15 mm/12 weeks	Empty	24.4
			Autologous bone	82
			Empty + Lactosorb	23.9
			DBM + Lactosorb	84
			CPC	13.1
			CPC + BMP	45.8

[39]	Rabbit/*n* = 10	6 mm	Ca-P	17
		6 mm	Empty	n.a.
		9 mm	Ca-P	18
		9 mm	Empty	n.a.
		15 mm	Ca-P	17
		15 mm/12 weeks	Empty	n.a.

[40]	Rabbit/*n* = 9 (4 defects/rabbit)	6 mm/4 weeks	PLGA	25.1
			PLGA/TCP	34.9
			BioOss	30.8
			empty	28.4

[41]	Rabbit/*n* = 33	6 mm/12 weeks	Polycaprolactone-TCP	32
		24 weeks	Polycaprolactone-TCP	25

[42]	Rabbit/*n* = 16	6 mm	6 mm empty	53.6
		8 mm	8 mm empty	41.8
		11 mm	11 mm empty	35.1
		15 mm/12 weeks	15 mm empty	20.1

[43]	Rabbit/*n* = 4/5	15 mm/6 weeks	TyrPC	16
			TyrPC + rhBMP-2	34
			TyrPC + CP	4
			BGS	4

[44]	Rabbit/*n* = 10	15 mm/12 weeks	CPC	14
			BC	21
			Empty	17.5
		6 weeks	BC + rh rhBMP-2	27

[45]	Rabbit/*n* = 4 for mPCL and empty, *n* = 6 for mPCL + BMPC and OB	15 mm/3 months	m-polycaprolactone	n.a.
			m-polycaprolactone + BMPC	4.7
			m-polycaprolactone + cOB	4.7
			empty	n.a.
		12 months	m-polycaprolactone	1.2
			m-polycaprolactone + BMPC	12.5
			m-polycaprolactone + cOB	12.5
			empty	1.2

DBM + Lactosorb (demineralized bone matrix), CPC (calcium phosphate cement), CPC + BMP (calcium phosphate cement + bone morphogenetic protein); Ca-P (calcium phosphate); PLGA (poly(lactide-coglycolide)), PLGA/TCP (PLGA/tricalcium phosphate), BioOss (bovine derived mineral); polycaprolactone-TCP (polycaprolactone-tricalcium phosphate); TyrPC (tyrosine-derived polycarbonate), TyrPC + rhBMP-2 (TyrPC + recombinant human bone morphogenetic protein-2), TyrPC + CP (TyrPC + calcium phosphate), BGS (bovine type-I collagen + tricalcium phosphate); CPC (calcium phosphate cement), BC (biocomposite = lysine derived polyurethane and allograft), BC + rhBMP-2 (BC + recombinant human bone morphogenetic protein-2); m-polycaprolactone (medical grade polycaprolactone), m-polycaprolactone + BMPC (m-polycaprolactone + bone marrow derived mesenchymal progenitor cells), m-polycaprolactone + cOB (m-polycaprolactone + calvarial osteoblasts).
